# Adverse neurological events associated with bispecific T cell engagers in oncology: a real-world study

**DOI:** 10.3389/fphar.2026.1819689

**Published:** 2026-05-21

**Authors:** Qianqian Cui, Shuangqi Gao, Hongyu Liu, Haiyun Zhou, Yunxiao Li

**Affiliations:** 1 Department of Pharmacy, The First College of Clinical Medical Science, China Three Gorges University and Yichang Central People’s Hospital, Yichang, China; 2 Department of Neurosurgery, Third Affiliated Hospital of Sun Yat-sen University, Guangzhou, Guangdong, China; 3 Department of Pharmacy, Tongji Hospital, Tongji Medical College, Huazhong University of Science and Technology, Wuhan, China

**Keywords:** bispecific T cell engager, cancer, FAERS, immune effector cell-associated neurotoxicity syndrome, NSTS

## Abstract

**Background:**

With the increasing use of bispecific T cell engagers (BiTEs) in anti-tumor immunotherapy, the associated adverse reactions pose significant challenges to clinical application. Nervous system toxicity (NST) is one of the notable adverse events associated with this class of drugs. This study aims to provide a comprehensive analysis of BiTE-induced nervous system adverse events. Improving the diagnosis and monitoring of these adverse events is crucial for the early identification and treatment of NSTs.

**Methods:**

We utilized the FAERS database to analyze NSTs associated with BiTEs reported between January 2004 and September 2025. Positive safety signals of the drugs were assessed using four commonly applied disproportionality analysis methods. In addition, the time to onset of drug-induced adverse reactions was evaluated.

**Results:**

A total of 15,558 patients who developed NSTs during BiTE therapy were included in this study. The average age at NST onset was 51.12 ± 24.44 years. The incidence was 21.19% higher in men than in women (6,139 men vs. 5,065 women), with the United States reporting the highest proportion of cases (50.68%). Among the BiTEs analyzed, blinatumomab accounted for the highest number of reported cases (49.63%). The NSTs observed include immune effector cell-associated neurotoxicity syndrome and other forms of neurotoxicity. Tremor, seizures, and nervous system disorders were reported more frequently with blinatumomab. In the comparative analysis of drug induction time, glofitamab exhibited the longest induction time [82.00 days (range, 1∼1190)], whereas tarlatamab showed the shortest time [16.70 days (range, 1∼141)].

**Conclusion:**

BiTEs have the potential to induce significant adverse events within the central nervous system and may exacerbate pre-existing conditions. Given the increasing utilization of BiTEs, it is essential to integrate resources such as the FAERS database to effectively monitor adverse reactions associated with these novel therapeutic agents.

## 1 Introduction

Cancer treatment has advanced substantially over the last decade, particularly with the advent of immunomodulatory therapies that enhance host anti-tumor immunity. Unlike traditional radiotherapy and chemotherapy, immunotherapy aims to eliminate tumor cells by harnessing the body’s own immune system. This approach has led to the development of numerous immunotherapeutic agents that are now available on the market. Among these, bispecific T cell engagers (BiTEs) have gained considerable attention and demonstrated great promise. BiTEs represent a transformative immunotherapy modality by effectively bridging the gap between T cell cytotoxicity and tumor antigen specificity. These engineered molecules simultaneously bind CD3 on T cells and tumor-associated antigens (TAAs) on cancer cells, thereby facilitating targeted immune activation and tumor cell lysis (Iwahori et al., 2015). Owing to their specificity and efficacy, BiTEs have become an attractive strategy for cancer treatment. Consequently, BiTEs targeting multiple tumor-associated antigens have been developed and achieved remarkable outcomes in clinical trials.

As of May 2025, the food and drug administration (FDA) has approved nine BiTE drugs, seven of which are indicated for treating hematological malignancies—blinatumomab, elranatamab, epcoritamab, glofitamab, mosunetuzumab, talquetamab, and teclistamab; the remaining two—tarlatamab and tebentafusp—are indicated for the treatment of solid tumors. Notably, blinatumomab has garnered significant attention as it was the first FDA-approved BiTE for clinical use in treating hematological malignancies. It targets CD19 and CD3, and is currently approved for the treatment of relapsed, refractory, and minimal residual disease (MRD)-positive B-cell acute lymphoblastic leukemia (BCP-ALL) in both adults and children. A Phase III clinical trial demonstrated that, compared to chemotherapy alone, the addition of blinatumomab to consolidation chemotherapy significantly improved overall survival in adult patients with MRD-negative remission from BCP-ALL (Litzow et al., 2024), with similar results observed in pediatric patients as well (Gupta et al., 2025). Tarlatamab is the first FDA-approved BiTE drug for the treatment of solid tumors (Ahn et al., 2023). This bispecific T-cell engager targets delta-like ligand 3 and has demonstrated durable anticancer activity with a manageable safety profile in previously treated patients with small cell lung cancer (SCLC), as evidenced in the DeLLphi-300 Phase I and DeLLphi-301 Phase II trials (Ahn et al., 2023; Paz-Ares et al., 2023). Furthermore, a Phase III trial reported that treatment with tarlatamab improved overall survival compared with chemotherapy in patients with SCLC whose disease progressed during or after platinum-based chemotherapy (Mountzios et al., 2025). Collectively, these findings underscore the considerable advantages of BiTEs in tumor treatment.

As with other anticancer drugs, adverse reactions associated with BiTEs can often lead to treatment interruption due to poor tolerability. Previous studies have reported that BiTEs may induce cytokine release syndrome (CRS), cardiovascular adverse reactions, anemia, infections, and diarrhea, among other side effects (Litzow et al., 2024; Labanca et al., 2025; Thieblemont et al., 2024). Furthermore, analyses of the FDA Adverse Event Reporting System (FAERS) database have revealed hepatobiliary toxicities associated with BiTE drugs, including conditions not documented in product labeling, such as ascites, hepatobiliary diseases, graft-versus-host disease after liver transplant, and veno-occlusive liver disease (Hu et al., 2025). Multiple clinical trials have also reported nervous system toxicity (NST) during BiTE treatment. Research indicates that BiTEs may lead to immune effector cell-associated neurotoxicity syndrome (ICANS) and other neurotoxic effects, with blinatumomab in particular being associated with epilepsy-related adverse reactions and cerebellar disorders (Bargou et al., 2008; Topp et al., 2011). The adverse reactions of these drugs can also vary depending on factors such as race, age, and dosage (Mocquot et al., 2022).

Given the inherent limitations of clinical trials—such as insufficient sample sizes and racial differences—the association between BiTEs and NSTs remains incompletely understood. Consequently, pharmacovigilance serves as a crucial method for uncovering post-marketing drug–adverse event (AE) relationships. FAERS constitutes the database for FDA’s post-marketing monitoring program for drugs and therapeutic biologics, collecting reports of AEs and medication errors. Therefore, utilizing FAERS data is essential for identifying pharmacovigilance signals during the clinical trial phase. To date, no systematic analysis has been performed on NSTs associated with BiTE drugs. Therefore, this study employed the FAERS database to analyze neurological AEs related to BiTE therapy reported in recent years, thereby establishing a theoretical foundation for understanding potential NSTs in patients receiving BiTE drugs.

## 2 Data and methods

### 2.1 Data source

FAERS is a crucial pharmacovigilance database utilized by the U.S. FDA to monitor post-marketing safety signals associated with drugs and medical products. Established as a repository for spontaneous AE reports, FAERS collects and compiles data from healthcare professionals, consumers, manufacturers, and legal representatives through the FDA MedWatch program. The FAERS database comprises seven core tables: DEMO (demographics), DRUG (drug/biologic information), REAC (adverse events), OUTC (patient outcomes), RPSR (report sources), THER (therapy dates), and INDI (indications). These tables are interconnected by unique identifiers to facilitate integrated analyses. This study analyzed all nervous system adverse reactions associated with BiTE drugs reported between January 2004 and September 2025. Adverse reactions in FAERS are coded using “Preferred Terms” (PT) codes from the Medical Dictionary for Regulatory Activities (MedDRA) to ensure standardized and accurate event classification and analysis. Integration with MedDRA and standardizing AE coding through both PTs and System Organ Classes (SOCs) enables granular signal detection via disproportionality analyses—such as the reporting odds ratio (ROR) and Bayesian Confidence Propagation Neural Network (BCPNN)—which quantify associations between drugs and specific AEs.

### 2.2 Statistical analysis

Detection of AE signals in FAERS is typically performed using the disproportionality analysis method, which identifies safety signals by comparing the occurrence proportion of AEs between a specified drug and all other drugs. In this study, the ROR and proportional reporting ratio (PRR) were applied. Additionally, BCPNN and the multi-item gamma Poisson shrinker (MGPS) were employed to detect AE signals associated with BiTE drugs, as detailed in Tables 1, 2. The combined application of these methods improves the robustness of the results through cross-validation. The ROR value evaluates the risk level of a target drug–AE combination in comparison to other drug–AE combinations; a higher ROR value indicates a stronger signal, reflecting a stronger correlation between the drug and AE. The PRR identifies signals by calculating proportional differences in AE reports between the target drug and all other drugs. Both BCPNN and MGPS are Bayesian statistical approaches that evaluate the strength of associations using the information component and empirically adjusted geometric means. All statistical analysis and data mining were performed using R software (version 4.5.1).

**TABLE 1:** Four-grid table of disproportionality analysis method.

Item	Target adverse events	All other adverse events	Total
Target drugs	a	b	a + b
All other drugs	c	d	c + d
Total	a + c	b + d	a+b + c + d

A contingency table for the calculation formula of the proportion imbalance analysis.

**TABLE 2:** Principle of disproportionality analysis and standard of signal detection.

Methods	Calculation formula	Inclusion standard of positive signal
ROR	$\text{ROR}=\frac{\left(a/c\right)}{\left(b/d\right)}$	a ≥ 3 and 95%CI > 1
	$\text{SE}\left(\ln\text{ROR}\right)=\sqrt{\left(\frac{1}{a}+\frac{1}{b}+\frac{1}{c}+\frac{1}{d}\right)}$	
	$95\%\text{CI}=e^{\ln\left(\text{ROR}\right)\pm 1.96\sqrt{\frac{1}{a}+\frac{1}{b}+\frac{1}{c}+\frac{1}{d}}}$	
PRR	$\text{PRR}=\frac{a/\left(a+b\right)}{c/\left(c+d\right)}$	a ≥ 3 and 95%CI > 1
	$\text{SE}\left(\ln\text{PRR}\right)=\sqrt{\left(\frac{1}{a}-\frac{1}{a+b}+\frac{1}{c}-\frac{1}{c+d}\right)}$	
	$95\%\text{CI}=e^{\ln\left(\text{PRR}\right)\pm 1.96\sqrt{\left(\frac{1}{a}-\frac{1}{a+b}+\frac{1}{c}-\frac{1}{c+d}\right)}}$	
BCPNN	$\text{IC}=\log_{2}\frac{a\left(a+b+c+d\right)}{\left(a+b\right)\left(a+c\right)}$	1) No signal (-): IC_025_ ≤ 0 2) Low signal (+):0<IC_025_ ≤ 1.5 3) Medium signal (++):1.5<IC_025_ ≤ 3 4) High signal (+++): IC_025_ > 3
	${\text{IC}}_{025}=\log_{2}\frac{\left(a+\gamma 11\right)\left(a+b+c+d+\alpha \right)\left(a+b+c+d+\beta \right)}{\left(a+b+c+d+\gamma \right)\left(a+b+\alpha 1\right)\left(a+c+\beta 1\right)}$	
	$V\left(\text{IC}\right)=\frac{1}{{\left(\ln2\right)}^{2}}\left\{\left[\frac{\left(a+b+c+d\right)-a+\gamma -\gamma 11}{\left(a+\gamma 11\right)\left(1+a+b+c+d+\gamma \right)}\right]+\left[\frac{\left(a+b+c+d\right)-\left(a+b\right)+a-\alpha 1}{\left(a+b+\alpha 1\right)\left(1+a+b+c+d+\alpha \right)}\right]+\left[\frac{\left(a+b+c+d\right)-\left(a+c\right)+\beta -\beta 1}{\left(a+c+\beta 1\right)\left(1+a+b+c+d+\beta \right)}\right]\right\}$	
	$\gamma =\gamma 11\frac{\left(a+b+c+d+\alpha \right)\left(a+b+c+d+\beta \right)}{\left(a+b+\alpha 1\right)\left(a+c+\beta 1\right)}$	
	$\text{IC}-2\text{SD}=E\left(\text{IC}\right)-2\sqrt{V\left(\text{IC}\right)}$	
	Where α1 = β1 = 1; α = β = 2; γ11=1	
MGPS	$\text{EBGM}=\frac{a\left(a+b+c+d\right)}{\left(a+c\right)\left(a+b\right)}$	EBGM05 > 2 and a>0
	$\text{EBGM}05=e^{\ln\left(\text{EBGM}\right)-\sqrt[{2}]{1.64\left(\frac{1}{a}+\frac{1}{b}+\frac{1}{c}+\frac{1}{d}\right)}}$	

Abbreviation: ROR, reporting odds ratio; PRR, proportional reported ratio; BCPNN, bayesian confidence propagation neural network; MGPS, multi-item gamma Poisson shrinker; CI, confidence interval; IC, information component.

## 3 Results

### 3.1 Descriptive analysis

**FIGURE 1:**
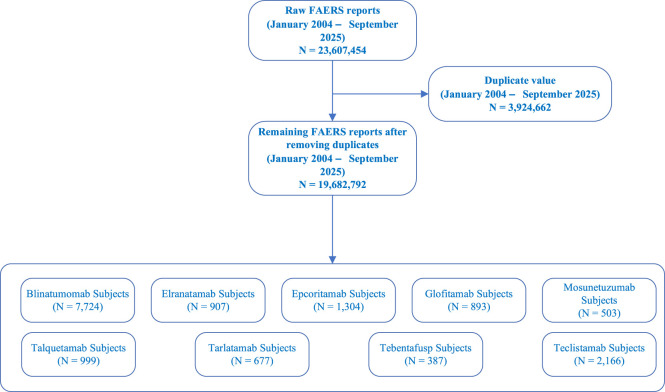
The FAERS database’s pipeline flowchart for screening BiTEs-related NSTs.

**FIGURE 2:**
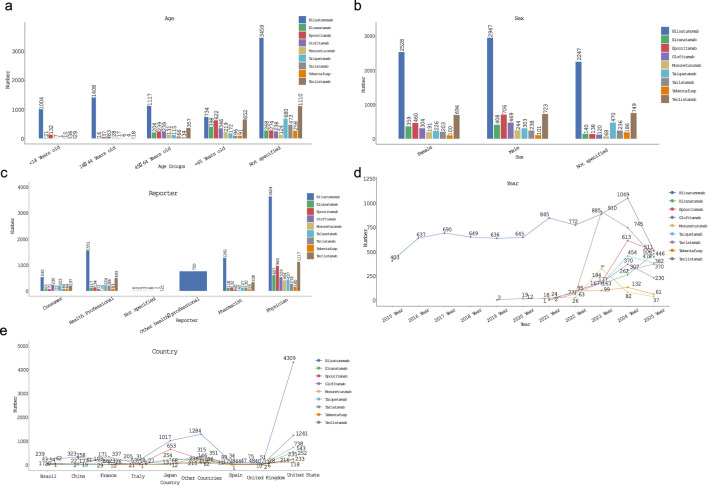
The characteristics of age, sex, reporter, year and country in BiTEs-related NSTs **(a)** the number of people of all ages who reported adverse neurological reactions; **(b)** the number of men and women reporting adverse neurological reactions; **(c)** the number of people reporting adverse neurological reactions; **(d)** the annual number of reported adverse reactions of the nervous system; **(e)** the number of reported cases of adverse neurological reactions in various countries.

A total of 23,607,454 reports from the FAERS database between January 2004 and September 2025 were analyzed. After deduplication, 19,682,792 reports remained, including 7,724 cases of blinatumomab, 907 cases of elranatamab, 1,304 cases of epcoritamab, 893 cases of glofitamab, 503 cases of mosunetuzumab, 999 cases of talquetamab, 677 cases of tarlatamab, 387 cases of tebentafusp, and 2,166 cases of teclistamab (Figure 1). The demographic and clinical characteristics of adverse drug events (ADEs) reported in the FAERS database, including age, weight, sex, reporting country, and outcomes, are summarized in Table 3; Figure 2. The results indicated that the average age at onset of adverse neurological reactions was 51.12 ± 24.44 years, with the highest incidence observed in individuals aged ≥45 years. The number of male patients was higher than that of female patients (6,139 males vs. 5,065 females), representing a 21.19% higher incidence in males. Reports were predominantly submitted by physicians, followed by other health professionals. Since 2022, the number of reported AEs has increased annually, with 3,021 cases reported in 2025. Among the reporting countries, the United States accounted for the highest number of reports (7,885 cases), followed by Japan (2,017 cases). Regarding outcome indicators, a total of 3,192 deaths were recorded, of which 1,398 cases (43.80%) were associated with blinatumomab. Additionally, 3,363 cases involved hospitalization due to initial or prolonged illness, with 1,367 cases (40.65%) linked to blinatumomab. Furthermore, a total of 5,072 cases were classified as other serious medical events, among which 2,686 cases (52.96%) were associated with blinatumomab.

**TABLE 3:** Demographic and clinical characteristics of BiTEs-related NST reports.

Variable	Total	Blinatumomab	Elranatamab	Epcoritamab	Glofitamab	Mosunetuzumab	Talquetamab	Tarlatamab	Tebentafusp	Teclistamab
	(N = 15,558)	(N = 7722)	(N = 907)	(N = 1304)	(N = 893)	(N = 503)	(N = 999)	(N = 677)	(N = 387)	(N = 2166)
Age	51.12 ± 24.44	38.59 ± 23.23	65.24 ± 15.95	60.25 ± 25.60	62.98 ± 14.45	65.69 ± 13.65	62.27 ± 17.13	54.09 ± 26.47	68.38 ± 10.46	65.58 ± 14.85
Weight	69.14 ± 22.19	64.24 ± 27.15	65.40 ± 17.16	64.39 ± 17.95	74.44 ± 20.50	77.64 ± 19.64	74.73 ± 20.73	67.73 ± 21.82	86.23 ± 28.05	72.05 ± 18.25
Sex										
Female	5065(32.56)	2528(32.74)	359(39.58)	460(35.28)	304(34.04)	191(37.97)	226(22.62)	203(29.99)	100(25.84)	694(32.04)
Male	6139(39.46)	2947(38.16)	408(44.98)	706(54.14)	469(52.52)	244(48.51)	303(30.33)	238(35.16)	101(26.10)	723(33.38)
Unknown	4354(27.99)	2247(29.10)	140(15.44)	138(10.58)	120(13.44)	68(13.52)	470(47.05)	236(34.86)	186(48.06)	749(34.58)
Reporter										
Consumer	1480(9.51)	530(6.86)	63(6.95)	71(5.44)	230(25.76)	22(4.37)	203(20.32)	80(11.82)	84(21.71)	197(9.10)
Health professional	2896(18.61)	1551(20.09)	117(12.90)	134(10.28)	71(7.95)	35(6.96)	224(22.42)	188(27.77)	83(21.45)	493(22.76)
Other health-professional	750(4.82)	750(9.71)	​	​	​	​	​	​	​	​
Pharmacist	2265(14.56)	1261(16.33)	118(13.01)	132(10.12)	60(6.72)	40(7.95)	127(12.71)	130(19.20)	59(15.25)	338(15.60)
Physician	8119(52.19)	3624(46.93)	607(66.92)	963(73.85)	529(59.24)	404(80.32)	437(43.74)	278(41.06)	160(41.34)	1117(51.57)
Unknown	48(0.31)	6(0.08)	2(0.22)	4(0.31)	3(0.34)	2(0.40)	8(0.80)	1(0.15)	1(0.26)	21(0.97)
Country										
Brazil	434(2.79)	239(3.10)	17(1.87)	41(3.14)	0(0)	20(3.98)	54(5.41)	1(0.15)	0(0)	62(2.86)
China	698(4.49)	323(4.18)	22(2.43)	2(0.15)	258(28.89)	17(3.38)	15(1.50)	0(0)	0(0)	61(2.82)
France	785(5.05)	171(2.21)	103(11.36)	29(2.22)	86(9.63)	23(4.57)	10(1.00)	0(0)	26(6.72)	337(15.56)
Italy	363(2.33)	205(2.65)	21(2.32)	53(4.06)	31(3.47)	11(2.19)	8(0.80)	1(0.15)	6(1.55)	27(1.25)
Japan	2017(12.96)	1017(13.17)	254(28.00)	653(50.08)	0(0)	13(2.58)	0(0)	68(10.04)	0(0)	12(0.55)
Other countries	2824(18.15)	1284(16.63)	215(23.70)	236(18.10)	315(35.27)	146(29.03)	119(11.91)	62(9.16)	96(24.81)	351(16.20)
Spain	281(1.81)	99(1.28)	11(1.21)	17(1.30)	34(3.81)	28(5.57)	44(4.40)	0(0)	1(0.26)	47(2.17)
United Kingdom	271(1.74)	75(0.97)	48(5.29)	40(3.07)	51(5.71)	10(1.99)	11(1.10)	2(0.30)	6(1.55)	28(1.29)
United state	7885(50.68)	4309(55.80)	216(23.81)	233(17.87)	118(13.21)	235(46.72)	738(73.87)	543(80.21)	252(65.12)	1241(57.29)
Outcome										
Death	3192(20.52)	1398(18.10)	227(25.03)	472(36.20)	284(31.80)	67(13.32)	79(7.91)	111(16.40)	66(17.05)	488(22.53)
Disability	42(0.27)	24(0.31)	2(0.22)	0(0)	1(0.11)	1(0.20)	5(0.50)	0(0)	1(0.26)	8(0.37)
Hospitalization - initial or prolonged	3363(21.62)	1367(17.70)	294(32.41)	260(19.94)	255(28.56)	260(51.69)	185(18.52)	114(16.84)	101(26.10)	527(24.33)
Life-threatening	597(3.84)	319(4.13)	29(3.20)	48(3.68)	39(4.37)	16(3.18)	29(2.90)	39(5.76)	8(2.07)	70(3.23)
Other serious (important medical event)	5072(32.60)	2686(34.78)	242(26.68)	461(35.35)	172(19.26)	69(13.72)	342(34.23)	275(40.62)	98(25.32)	727(33.56)
Required intervention to prevent permanent impairment/Damage	21(0.13)	3(0.04)	0(0)	0(0)	1(0.11)	0(0)	4(0.40)	1(0.15)	1(0.26)	11(0.51)
Unknown	3271(21.02)	1925(24.93)	113(12.46)	63(4.83)	141(15.79)	90(17.89)	355(35.54)	137(20.24)	112(28.94)	335(15.47)
Year										
2015 Year	403(2.59)	403(5.22)	0(0)	0(0)	0(0)	0(0)	0(0)	0(0)	0(0)	0(0)
2016 Year	637(4.09)	637(8.25)	0(0)	0(0)	0(0)	0(0)	0(0)	0(0)	0(0)	0(0)
2017 Year	690(4.44)	690(8.94)	0(0)	0(0)	0(0)	0(0)	0(0)	0(0)	0(0)	0(0)
2018 Year	649(4.17)	649(8.40)	0(0)	0(0)	0(0)	0(0)	0(0)	0(0)	0(0)	0(0)
2019 Year	638(4.10)	636(8.24)	0(0)	0(0)	2(0.22)	0(0)	0(0)	0(0)	0(0)	0(0)
2020 Year	676(4.35)	645(8.35)	0(0)	0(0)	19(2.13)	12(2.39)	0(0)	0(0)	0(0)	0(0)
2021 Year	890(5.72)	845(10.94)	0(0)	1(0.08)	18(2.02)	24(4.77)	0(0)	0(0)	0(0)	2(0.09)
2022 Year	1033(6.64)	772(10.00)	0(0)	0(0)	77(8.62)	26(5.17)	0(0)	0(0)	95(24.55)	63 2.91)
2023 Year	2907(18.68)	885(11.46)	167(18.41)	184(14.11)	177(19.82)	322(64.02)	163(16.32)	0(0)	99(25.58)	910(42.01)
2024 Year	4014(25.80)	1049(13.58)	262(28.89)	613(47.01)	370(41.43)	82(16.30)	454(45.45)	307(45.35)	132(34.11)	745(34.40)
2025 Year	3021(19.42)	511(6.62)	478(52.70)	506(38.80)	230(25.76)	37(7.36)	382(38.24)	370(54.65)	61(15.76)	446(20.59)

### 3.2 Signal spectrum of NSTs associated with BiTEs

**FIGURE 3:**
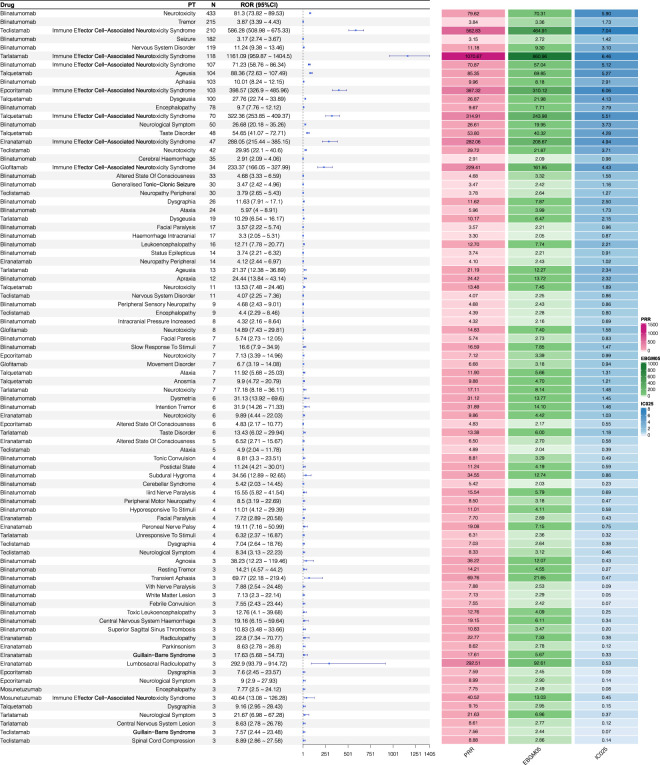
ROR, PRR, EBGM05, IC025 values and the number of reports of signal PTs for BiTEs-related NSTs. ROR, reporting odds ratio; 95% CI, 95% confidence interval; PRR, proportional reporting ratio; EBGM, empirical Bayesian geometric mean; EBGM05, the lower limit of 95% CI of EBGM; IC, information component; IC025, the lower limit of 95% CI of IC.

We employed four disproportionality methods in this study: ROR, PRR, EBGM05, and IC025. If all four algorithms yielded positive signals, the outcome was classified as positive. The signal values for NSTs associated with BiTEs are illustrated in Figure 3; Table 4. The results indicate that several drugs exhibited a high proportion of ICANS, with notably strong positive signals. The highest ROR was observed for tarlatamab (ROR: 1161.09; 95% CI: 959.87–1404.5), followed by teclistamab (ROR: 586.28; 95% CI: 508.98–675.33). The ROR value for epcoritamab was 398.57 (95% CI: 326.9–485.96), talquetamab was 322.36 (95% CI: 253.85–409.37), elranatamab was 288.05 (95% CI: 215.44–385.15), and glofitamab was 233.37 (95% CI: 166.05–327.99). Furthermore, NSTs were observed in several drugs. Among the BiTE drugs analyzed, blinatumomab accounted for the highest number of reported neurological adverse reactions. The five most frequently reported NSTs (exceeding 100 cases each) were: neurotoxicity (433 cases), tremor (215 cases), seizure (182 cases), nervous system disorder (119 cases), and ICANS (107 cases). For teclistamab, the most frequently reported NST was ICANS, followed by neurotoxicity. Tarlatamab was mainly associated with ICANS and dysgeusia, while talquetamab was associated with ageusia and dysgeusia. For epcoritamab, the most reported NST was ICANS, followed by neurotoxicity. Both elranatamab and glofitamab were predominantly associated with ICANS, whereas mosunetuzumab was associated with relatively fewer cases of neurotoxicity (three cases each of encephalopathy and ICANS). The positive signal results of the disproportionality analysis for neurological adverse reactions at the PT level are detailed in Table 4.

**TABLE 4:** Positive signal of adverse neurological reaction (PT) disproportionation analysis of drugs.

Drug	PT	ROR (95%CI)	PRR (95%CI)	MGPS (95%CI)	BCPNN (95%CI)	PRR (X^2^)
Blinatumomab	Agnosia	38.23(12.23–119.46)	38.22(37.08–39.36)	37.72(14.54–97.86)	5.24(3.56–6.91)	38.22(107.28)
Blinatumomab	Seizure	3.17(2.74–3.67)	3.15(3.01–3.3)	3.15(2.79–3.56)	1.65(-0.01–3.32)	3.15(267.6)
Blinatumomab	Ataxia	5.97(4–8.91)	5.96(5.56–6.36)	5.95(4.26–8.32)	2.57(0.91–4.24)	5.96(98.9)
Blinatumomab	Apraxia	24.44(13.84–43.14)	24.42(23.85–24.99)	24.22(15.05–38.97)	4.6(2.93–6.27)	24.42(267.23)
Blinatumomab	Aphasia	10.01(8.24–12.15)	9.96(9.77–10.16)	9.93(8.44–11.68)	3.31(1.65–4.98)	9.96(828.17)
Blinatumomab	Tremor	3.87(3.39–4.43)	3.84(3.71–3.98)	3.84(3.43–4.3)	1.94(0.28–3.61)	3.84(453.19)
Blinatumomab	Dysmetria	31.13(13.92–69.6)	31.12(30.31–31.92)	30.79(15.7–60.36)	4.94(3.27–6.61)	31.12(172.97)
Blinatumomab	Intention tremor	31.9(14.26–71.33)	31.89(31.08–32.69)	31.54(16.08–61.85)	4.98(3.31–6.65)	31.89(177.49)
Blinatumomab	Neurotoxicity	81.3(73.82–89.53)	79.62(79.52–79.71)	77.43(71.43–83.95)	6.27(4.61–7.94)	79.62(32,689.48)
Blinatumomab	Tonic convulsion	8.81(3.3–23.51)	8.81(7.83–9.79)	8.78(3.86–19.97)	3.13(1.47–4.8)	8.81(27.6)
Blinatumomab	Dysgraphia	11.63(7.91–17.1)	11.62(11.23–12)	11.57(8.38–15.98)	3.53(1.87–5.2)	11.62(251.3)
Blinatumomab	Facial paralysis	3.57(2.22–5.74)	3.57(3.09–4.04)	3.56(2.39–5.31)	1.83(0.17–3.5)	3.57(31.36)
Blinatumomab	Postictal state	11.24(4.21–30.01)	11.24(10.26–12.22)	11.2(4.92–25.47)	3.49(1.82–5.15)	11.24(37.17)
Blinatumomab	Resting tremor	14.21(4.57–44.2)	14.21(13.08–15.35)	14.14(5.47–36.55)	3.82(2.15–5.49)	14.21(36.66)
Blinatumomab	Facial paresis	5.74(2.73–12.05)	5.74(5–6.48)	5.73(3.08–10.66)	2.52(0.85–4.19)	5.74(27.34)
Blinatumomab	Encephalopathy	9.7(7.76–12.12)	9.67(9.45–9.89)	9.64(8–11.61)	3.27(1.6–4.93)	9.67(604.36)
Blinatumomab	Subdural hygroma	34.56(12.89–92.65)	34.55(33.57–35.54)	34.14(14.96–77.92)	5.09(3.42–6.77)	34.55(128.74)
Blinatumomab	Transient aphasia	69.77(22.18–219.4)	69.76(68.61–70.9)	68.08(26.1–177.57)	6.09(4.4–7.78)	69.76(198.35)
Blinatumomab	Nervous system disorder	11.24(9.38–13.46)	11.18(11–11.36)	11.14(9.58–12.96)	3.48(1.81–5.14)	11.18(1099.29)
Blinatumomab	Slow response to stimuli	16.6(7.9–34.9)	16.59(15.85–17.34)	16.5(8.86–30.73)	4.04(2.38–5.71)	16.59(101.98)
Blinatumomab	Cerebellar syndrome	5.42(2.03–14.45)	5.42(4.44–6.4)	5.41(2.38–12.29)	2.44(0.77–4.1)	5.42(14.38)
Blinatumomab	Vith nerve paralysis	7.88(2.54–24.48)	7.88(6.75–9.02)	7.86(3.05–20.3)	2.98(1.31–4.64)	7.88(17.98)
Blinatumomab	Status epilepticus	3.74(2.21–6.32)	3.74(3.22–4.26)	3.74(2.41–5.79)	1.9(0.24–3.57)	3.74(28.06)
Blinatumomab	Iiird nerve paralysis	15.55(5.82–41.54)	15.54(14.56–16.53)	15.46(6.79–35.19)	3.95(2.28–5.62)	15.54(54.13)
Blinatumomab	White matter lesion	7.13 (2.3–22.14)	7.13 (6–8.26)	7.11 (2.76–18.36)	2.83 (1.16–4.5)	7.13 (15.77)
Blinatumomab	Haemorrhage intracranial	3.3 (2.05–5.31)	3.3 (2.82–3.77)	3.3 (2.21–4.91)	1.72 (0.05–3.39)	3.3 (27.22)
Blinatumomab	Neurological symptom	26.68 (20.18–35.26)	26.61 (26.34–26.89)	26.37 (20.88–33.3)	4.72 (3.05–6.39)	26.61 (1221.06)
Blinatumomab	Febrile convulsion	7.55 (2.43–23.44)	7.55 (6.41–8.68)	7.53 (2.92–19.44)	2.91 (1.24–4.58)	7.55 (17)
Blinatumomab	Leukoencephalopathy	12.71 (7.78–20.77)	12.7 (12.21–13.19)	12.65 (8.38–19.08)	3.66 (1.99–5.33)	12.7 (171.7)
Blinatumomab	Cerebral haemorrhage	2.91 (2.09–4.06)	2.91 (2.58–3.24)	2.91 (2.2–3.84)	1.54 (-0.13–3.21)	2.91 (43.85)
Blinatumomab	Intracranial pressure increased	4.32 (2.16–8.64)	4.32 (3.62–5.01)	4.31 (2.41–7.71)	2.11 (0.44–3.77)	4.32 (20.36)
Blinatumomab	Toxic leukoencephalopathy	12.76 (4.1–39.68)	12.76 (11.63–13.89)	12.71 (4.92–32.83)	3.67 (2–5.34)	12.76 (32.37)
Blinatumomab	Generalised tonic-clonic seizure	3.47 (2.42–4.96)	3.47 (3.11–3.82)	3.46 (2.57–4.67)	1.79 (0.13–3.46)	3.47 (52.57)
Blinatumomab	Peripheral motor neuropathy	8.5 (3.19–22.69)	8.5 (7.52–9.48)	8.48 (3.73–19.27)	3.08 (1.42–4.75)	8.5 (26.39)
Blinatumomab	Altered state of consciousness	4.68 (3.33–6.59)	4.68 (4.34–5.02)	4.67 (3.51–6.22)	2.22 (0.56–3.89)	4.68 (95.29)
Blinatumomab	Hyporesponsive to stimuli	11.01 (4.12–29.39)	11.01 (10.03–11.99)	10.97 (4.82–24.94)	3.46 (1.79–5.12)	11.01 (36.25)
Blinatumomab	Peripheral sensory neuropathy	4.68 (2.43–9.01)	4.68 (4.03–5.33)	4.67 (2.7–8.08)	2.22 (0.56–3.89)	4.68 (26.01)
Blinatumomab	Central nervous system haemorrhage	19.16 (6.15–59.64)	19.15 (18.02–20.29)	19.03 (7.36–49.22)	4.25 (2.58–5.92)	19.15 (51.27)
Blinatumomab	Immune effector cell-associated neurotoxicity syndrome	71.23 (58.76–86.34)	70.87 (70.67–71.06)	69.13 (58.86–81.21)	6.11 (4.44–7.78)	70.87 (7188.08)
Blinatumomab	Superior sagittal sinus thrombosis	10.83 (3.48–33.66)	10.83 (9.7–11.96)	10.79 (4.18–27.87)	3.43 (1.76–5.1)	10.83 (26.66)
Elranatamab	Facial paralysis	7.72 (2.89–20.58)	7.7 (6.72–8.68)	7.7 (3.39–17.5)	2.95 (1.28–4.61)	7.7 (23.33)
Elranatamab	Neurotoxicity	9.89 (4.44–22.03)	9.86 (9.06–10.66)	9.86 (5.04–19.28)	3.3 (1.63–4.97)	9.86 (47.77)
Elranatamab	Radiculopathy	22.8 (7.34–70.77)	22.77 (21.64–23.9)	22.75 (8.82–58.7)	4.51 (2.84–6.18)	22.77 (62.38)
Elranatamab	Parkinsonism	8.63 (2.78–26.8)	8.62 (7.49–9.75)	8.62 (3.34–22.24)	3.11 (1.44–4.78)	8.62 (20.21)
Elranatamab	Guillain-barre syndrome	17.63 (5.68–54.73)	17.61 (16.48–18.74)	17.6 (6.82–45.4)	4.14 (2.47–5.8)	17.61 (46.97)
Elranatamab	Peroneal nerve palsy	19.11 (7.16–50.99)	19.08 (18.1–20.06)	19.07 (8.39–43.34)	4.25 (2.59–5.92)	19.08 (68.48)
Elranatamab	Neuropathy peripheral	4.12 (2.44–6.97)	4.1 (3.58–4.63)	4.1 (2.64–6.37)	2.04 (0.37–3.7)	4.1 (32.92)
Elranatamab	Altered state of consciousness	6.52 (2.71–15.67)	6.5 (5.63–7.38)	6.5 (3.12–13.55)	2.7 (1.03–4.37)	6.5 (23.29)
Elranatamab	Immune effector cell-associated neurotoxicity syndrome	288.05 (215.44–385.15)	282.06 (281.77–282.34)	279 (218.8–355.77)	8.12 (6.46–9.79)	282.06 (13,020.72)
Elranatamab	Lumbosacral radiculopathy	292.9 (93.79–914.72)	292.51 (291.37–293.65)	289.22 (111.53–749.99)	8.18 (6.5–9.85)	292.51 (861.72)
Epcoritamab	Neurotoxicity	7.13 (3.39–14.96)	7.12 (6.37–7.86)	7.11 (3.82–13.23)	2.83 (1.16–4.5)	7.12 (36.78)
Epcoritamab	Dysgraphia	7.6 (2.45–23.57)	7.59 (6.46–8.72)	7.59 (2.94–19.57)	2.92 (1.26–4.59)	7.59 (17.16)
Epcoritamab	Neurological symptom	9 (2.9–27.93)	8.99 (7.86–10.13)	8.99 (3.49–23.19)	3.17 (1.5–4.84)	8.99 (21.31)
Epcoritamab	Altered state of consciousness	4.83 (2.17–10.77)	4.83 (4.03–5.63)	4.83 (2.47–9.43)	2.27 (0.6–3.94)	4.83 (18.21)
Epcoritamab	Immune effector cell-associated neurotoxicity syndrome	398.57 (326.9–485.96)	387.32 (387.13–387.52)	378.11 (320.32–446.33)	8.56 (6.9–10.23)	387.32 (38,745.12)
Glofitamab	Neurotoxicity	14.89 (7.43–29.81)	14.83 (14.14–15.52)	14.82 (8.29–26.51)	3.89 (2.22–5.56)	14.83 (103.16)
Glofitamab	Movement disorder	6.7 (3.19–14.08)	6.68 (5.94–7.42)	6.68 (3.59–12.43)	2.74 (1.07–4.41)	6.68 (33.83)
Glofitamab	Immune effector cell-associated neurotoxicity syndrome	233.37 (166.05–327.99)	229.41 (229.08–229.75)	227.62 (171.21–302.61)	7.83 (6.16–9.5)	229.41 (7671.93)
Mosunetuzumab	Encephalopathy	7.77 (2.5–24.12)	7.75 (6.62–8.88)	7.74 (3–19.99)	2.95 (1.28–4.62)	7.75 (17.63)
Mosunetuzumab	Immune effector cell-associated neurotoxicity syndrome	40.64 (13.08–126.28)	40.52 (39.39–41.65)	40.49 (15.68–104.56)	5.34 (3.67–7.01)	40.52 (115.56)
Talquetamab	Ataxia	11.92 (5.68–25.03)	11.9 (11.16–12.64)	11.89 (6.39–22.12)	3.57 (1.9–5.24)	11.9 (69.83)
Talquetamab	Anosmia	9.9 (4.72–20.79)	9.88 (9.14–10.62)	9.88 (5.31–18.38)	3.3 (1.64–4.97)	9.88 (55.87)
Talquetamab	Ageusia	88.36 (72.63–107.49)	85.35 (85.16–85.54)	84.97 (72.12–100.12)	6.41 (4.74–8.08)	85.35 (8634.46)
Talquetamab	Neurotoxicity	13.53 (7.48–24.46)	13.48 (12.89–14.07)	13.48 (8.21–22.12)	3.75 (2.09–5.42)	13.48 (127.09)
Talquetamab	Taste disorder	54.65 (41.07–72.71)	53.8 (53.51–54.08)	53.65 (42.24–68.13)	5.75 (4.08–7.41)	53.8 (2480.82)
Talquetamab	Dysgraphia	9.16 (2.95–28.43)	9.15 (8.02–10.28)	9.15 (3.55–23.6)	3.19 (1.53–4.86)	9.15 (21.78)
Talquetamab	Dysgeusia	27.76 (22.74–33.89)	26.87 (26.68–27.06)	26.84 (22.71–31.71)	4.75 (3.08–6.41)	26.87 (2490.47)
Talquetamab	Immune effector cell-associated neurotoxicity syndrome	322.36 (253.85–409.37)	314.91 (314.68–315.15)	309.83 (253.69–378.39)	8.28 (6.61–9.94)	314.91 (21,550.85)
Tarlatamab	Ageusia	21.37 (12.38–36.89)	21.19 (20.65–21.73)	21.18 (13.41–33.45)	4.4 (2.74–6.07)	21.19 (250.07)
Tarlatamab	Neurotoxicity	17.18 (8.18–36.11)	17.11 (16.37–17.85)	17.1 (9.19–31.83)	4.1 (2.43–5.76)	17.11 (106.14)
Tarlatamab	Taste disorder	13.43 (6.02–29.94)	13.38 (12.58–14.17)	13.37 (6.84–26.16)	3.74 (2.07–5.41)	13.38 (68.7)
Tarlatamab	Dysgeusia	10.29 (6.54–16.17)	10.17 (9.72–10.62)	10.17 (6.96–14.85)	3.35 (1.68–5.01)	10.17 (157.24)
Tarlatamab	Unresponsive to stimuli	6.32 (2.37–16.87)	6.31 (5.33–7.29)	6.31 (2.77–14.34)	2.66 (0.99–4.32)	6.31 (17.87)
Tarlatamab	Neurological symptom	21.67 (6.98–67.28)	21.63 (20.5–22.76)	21.61 (8.38–55.78)	4.43 (2.77–6.1)	21.63 (58.99)
Tarlatamab	Central nervous system lesion	8.63 (2.78–26.78)	8.61 (7.48–9.74)	8.61 (3.34–22.21)	3.11 (1.44–4.77)	8.61 (20.18)
Tarlatamab	Immune effector cell-associated neurotoxicity syndrome	1161.09 (959.87–1404.5)	1070.67 (1070.5–1070.85)	1041.45 (888.14–1221.24)	10.02 (8.36–11.69)	1070.67 (122,668.18)
Teclistamab	Ataxia	4.9 (2.04–11.78)	4.89 (4.02–5.77)	4.89 (2.35–10.19)	2.29 (0.62–3.96)	4.89 (15.49)
Teclistamab	Neurotoxicity	29.95 (22.1–40.6)	29.72 (29.42–30.02)	29.64 (22.99–38.23)	4.89 (3.22–6.56)	29.72 (1162.89)
Teclistamab	Dysgraphia	7.04 (2.64–18.76)	7.03 (6.05–8.01)	7.03 (3.09–15.97)	2.81 (1.15–4.48)	7.03 (20.69)
Teclistamab	Encephalopathy	4.4 (2.29–8.46)	4.39 (3.74–5.04)	4.39 (2.54–7.59)	2.13 (0.47–3.8)	4.39 (23.57)
Teclistamab	Nervous system disorder	4.07 (2.25–7.36)	4.07 (3.47–4.66)	4.06 (2.48–6.67)	2.02 (0.36–3.69)	4.07 (25.43)
Teclistamab	Neurological symptom	8.34 (3.13–22.23)	8.33 (7.35–9.31)	8.33 (3.67–18.92)	3.06 (1.39–4.72)	8.33 (25.79)
Teclistamab	Guillain-barre syndrome	7.57 (2.44–23.48)	7.56 (6.43–8.7)	7.56 (2.93–19.5)	2.92 (1.25–4.59)	7.56 (17.08)
Teclistamab	Spinal cord compression	8.89 (2.86–27.58)	8.88 (7.75–10.02)	8.88 (3.44–22.9)	3.15 (1.48–4.82)	8.88 (20.97)
Teclistamab	Neuropathy peripheral	3.79 (2.65–5.43)	3.78 (3.42–4.14)	3.78 (2.8–5.1)	1.92 (0.25–3.58)	3.78 (61.37)
Teclistamab	Immune effector cell-associated neurotoxicity syndrome	586.28 (508.98–675.33)	562.83 (562.69–562.96)	535.52 (475.76–602.77)	9.06 (7.4–10.73)	562.83 (112,057.27)

**FIGURE 4:**
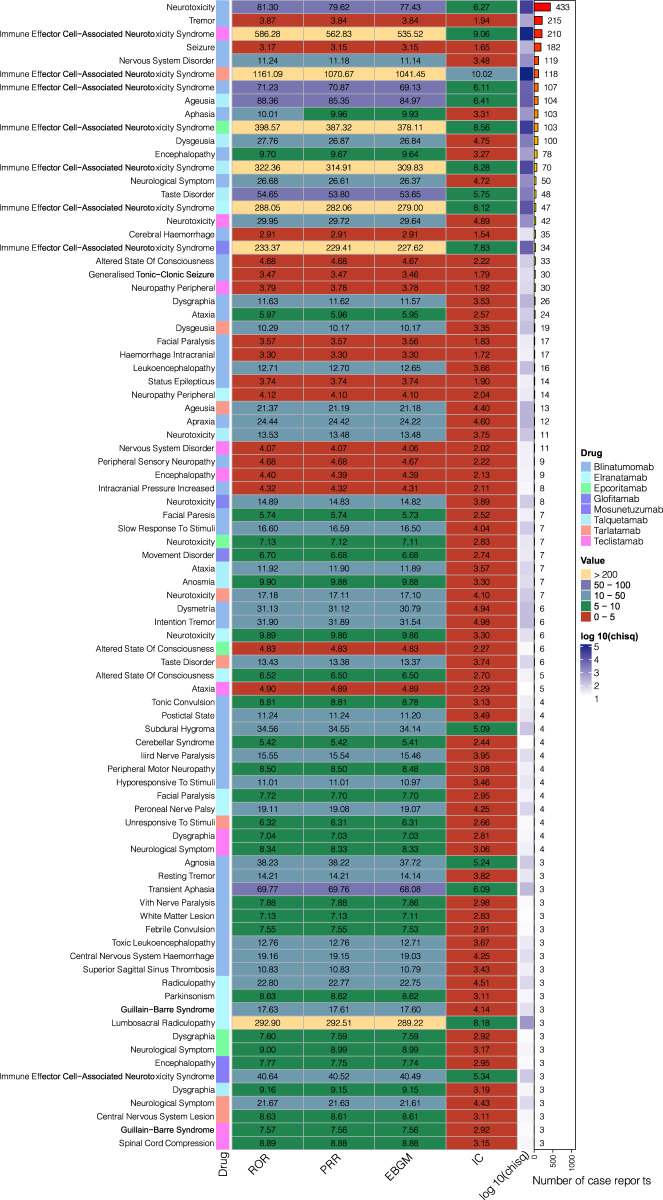
Heatmap of positive signal disproportionation analysis.

Figure 4 presents an analysis of the positive signals indicating NSTs associated with several drugs, utilizing heat maps for visualization. These findings reveal that the top five PTs related to NSTs are neurotoxicity, tremor, ICANS, seizure, and nervous system disorder. Conversely, the bottom five PTs include dysgraphia, neurological symptom, central nervous system lesion, Guillain-Barré syndrome, and spinal cord compression. Additionally, an increase in reported cases of ageusia and dysgeusia was noted. Eight drugs exhibited various neurological toxicity PTs that showed positive signals. Notably, while the signal values for tebentafusp concerning different neurological toxicity PTs were not positive, positive signals were identified in the analysis of neurological adverse reactions (SOC) through disproportionality analysis. The results of this analysis for the neurological adverse reactions (SOC) associated with the drugs are detailed in Table 5.

**TABLE 5:** Positive signal of adverse neurological reaction (SOC) disproportionation analysis of drugs.

Drug name	ROR (95%CI)	PRR (95%CI)	MGPS (95%CI)	BCPNN (95%CI)	PRR (X^2^)
Blinatumomab	1.67 (1.61–1.74)	1.58 (1.55–1.62)	1.58 (1.53–1.64)	0.66 (-1–2.33)	1.58 (649.97)
Elranatamab	1 (0.86–1.16)	1 (0.87–1.14)	1 (0.88–1.13)	0 (-1.66–1.67)	1 (0)
Epcoritamab	0.77 (0.67–0.87)	0.78 (0.66–0.91)	0.78 (0.7–0.87)	−0.35 (-2.02 to 1.31)	0.78 (15.8)
Glofitamab	0.59 (0.48–0.71)	0.61 (0.42–0.8)	0.61 (0.51–0.72)	−0.72 (-2.39 to 0.95)	0.61 (28.4)
Mosunetuzumab	0.69 (0.53–0.9)	0.71 (0.46–0.95)	0.71 (0.57–0.88)	−0.5 (-2.17 to 1.17)	0.71 (7.79)
Talquetamab	2.07 (1.87–2.28)	1.9 (1.81–1.98)	1.9 (1.75–2.06)	0.92 (-0.74–2.59)	1.9 (222.69)
Tarlatamab	2.15 (1.88–2.46)	1.96 (1.85–2.07)	1.96 (1.75–2.2)	0.97 (-0.7–2.64)	1.96 (128.46)
Tebentafusp	0.52 (0.4–0.68)	0.54 (0.29–0.8)	0.54 (0.44–0.68)	−0.88 (-2.55 to 0.79)	0.54 (23.73)
Teclistamab	1.32 (1.21–1.44)	1.29 (1.21–1.36)	1.29 (1.2–1.38)	0.36 (-1.3–2.03)	1.29 (39.68)

The p-value represents the statistical test value from the chi-square test in the PRR, algorithm. All of the above drugs meet the positive signal screening criteria for disproportionality analysis.

Abbreviations: BCPNN, bayesian confidence propagation neural network; MGPS, multi-item gamma Poisson shrinker; PRR, proportional reported ratio; ROR, reporting odds ratio; CI, confidence interval.

### 3.3 Time to onset of adverse events

**FIGURE 5:**
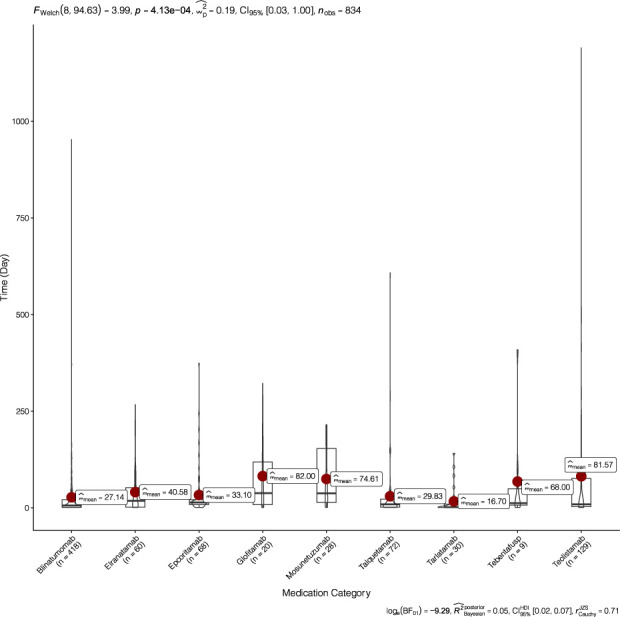
Time to onset of BiTEs-related NSTs.

Figure 5 illustrates the onset times of NSTs induced by nine marketed BiTEs. Among these, tarlatamab exhibited the shortest induction time of 16.70 (1∼141) days, followed by blinatumomab at 27.14 (1∼953) days. Conversely, glofitamab demonstrated the longest drug-induced duration at 82 (1∼322) days, closely followed by teclistamab, which had an induction time of 81.57 (1∼1190) days. Notably, blinatumomab had the largest sample size of 418 cases for the induction time of adverse reactions, while teclistamab followed with 129 cases. In contrast, tebentafusp presented the smallest sample size, comprising only nine cases.

## 4 Discussion

BiTEs represent a groundbreaking class of immunotherapies that redirect T cells to target tumor-associated antigens, demonstrating remarkable efficacy in hematological malignancies, such as B-ALL and multiple myeloma. However, their clinical application is often complicated by neurological toxicities, including ICANS and other forms of neurotoxicity. To date, the overall incidence of neurological adverse reactions in patients with hematological malignancies and solid tumors treated with BiTE drugs has not been systematically reported. This study collected data from January 2004 to September 2025 through the FAERS database and systematically analyzed the neurological side effects associated with nine BiTE drugs currently approved for marketing by the FDA. The findings of this study provide insights into potential neurological side effects for patients using BiTE drugs and offer directions for monitoring early signs of NSTs.

BiTE drugs may cause severe toxicity related to T cell activation. According to existing studies, the most common AE associated with BiTE drugs is cytokine release syndrome (CRS), which is an acute systemic inflammatory response primarily characterized by fever (Hutchings et al., 2021; Crombie et al., 2024). Evidence indicates that the severity of symptoms in patients with CRS varies, with most cases being mild. The likelihood of developing high-grade CRS is relatively low (Crombie et al., 2024). Furthermore, neurologic toxicities represent another common AE, with acute manifestations including headache and dizziness. Among these, ICANS is the most prevalent neurologic toxicity, and these symptoms may occur concurrently with CRS. Additionally, infections, particularly urinary tract infections and pneumonia, have also been reported. Other adverse reactions, such as tumor flare reactions and cytopenias, have been documented (Crombie et al., 2024). Therefore, it is crucial to closely monitor patient manifestations during clinical use, ensuring early detection and timely intervention.

Our study found that adverse reactions related to ICANS occurred in several BiTE drugs. ICANS is characterized by symptoms associated with neuropsychiatric disorders. Although most patients can gradually recover tolerance after treatment, some may develop severe and even life-threatening ICANS (Rubin et al., 2019; Gust et al., 2017). Studies indicate that 9%–26% of patients treated with blinatumomab experience severe neurological adverse events (Viardot et al., 2016; Gokbuget et al., 2018; Topp et al., 2015). Cases of severe ICANS induced by BiTEs drugs are relatively rare, although the incidence and severity of ICANS are higher in patients with untreated brain metastases (Hu et al., 2025). According to relevant guidelines and recommendations, treatment methods for ICANS vary slightly. Grade 1 generally does not necessitate special treatment. For grade 2, administration of 10 mg of dexamethasone every 12 h is required. Grade 3 necessitates 10 mg of dexamethasone every 6 h, while grade 4 requires 20 mg of dexamethasone every 6 h. Adjustments should be made based on the therapeutic effect (Ludwig et al., 2023). Despite the relatively high incidence of ICANS among BiTE drugs, the underlying pathophysiological mechanisms remain unclear. The brain is protected by the blood-brain barrier; however, studies have demonstrated that BiTE drugs can be detected in cerebrospinal fluid, along with activated T and B cells (Klinger et al., 2020). The initial adhesion of BiTE-activated T cells to the endothelial layer of blood vessels is a critical step in their transmigration across the blood-brain barrier. Once T cells enter the central nervous system, they can eliminate resident B cells, leading to an increase in inflammatory factors (Klinger et al., 2020). Concurrently, the heightened production of pro-inflammatory cytokines may activate endothelial cells, resulting in increased permeability of the blood-brain barrier and elevated cytokine levels in cerebrospinal fluid (Markouli et al., 2023). This may contribute to NSTs associated with BiTE drugs. In addition to ICANS, BiTE drugs are associated with a relatively high likelihood of neurotoxicity, with tremors and seizures being common adverse effects associated with blinatumomab.

Given the frequent occurrence of NSTs associated with BiTE drugs, techniques aimed at improving their safety profile are continuously being developed. Innovative strategies to mitigate BiTE-associated NSTs are currently under active investigation. One promising approach involves engineering BiTEs with reduced CD3 affinity, which tempers excessive T cell activation while preserving anti-tumor efficacy (Dao et al., 2015). Preclinical studies demonstrated that a lower CD3 binding affinity correlates with diminished cytokine release and reduced neurotoxicity, without compromising tumor cell lysis (Yang et al., 2024). Another advancement is the development of switchable BiTE platforms, such as supramolecular T cell nanoengagers, which can be disassembled using small-molecule drugs like amantadine to rapidly halt off-tumor toxicity and neuroinflammation (Cirstea et al., 2025). Furthermore, the integration of sialidase enzymes into BiTE constructs has shown promise in enhancing immunological synapse formation while simultaneously reducing systemic cytokine release, thereby attenuating neurotoxicity (Leithner et al., 2025).

According to the available data, the average age at onset of BiTE-associated NSTs was 51.12 ± 24.44 years. Notably, blinatumumab exhibits a higher reporting rate of NST in individuals under 65 years of age, whereas the reporting rate is the lowest among those over 65 years. This suggests that individuals under 65 are at a higher risk of experiencing NSTs when treated with blinatumumab. Consequently, it is crucial to monitor NSTs in patients under 65 years during the clinical administration of blinatumumab. In contrast, the other eight BiTEs reported a higher incidence of NST in patients over 45 years, with a lower reporting rate in those under 44 years. It is essential to recognize that these drugs pose a considerable NST risk in individuals over 45, warranting close monitoring during clinical use. Overall, the incidence of NSTs appears to be lower in older patients compared to younger counterparts.

We also analyzed the time to onset of NSTs following BiTE treatment. The onset of NSTs associated with blinatumumab occurs at an average of 27.14 days (range, 1∼953), which aligns closely with the median onset time of 20.85 days reported for NSTs. Furthermore, the onset time for fatal NSTs is slightly longer than that for non-fatal NSTs (Gao et al., 2025). The induction times for different BiTEs vary significantly; for instance, glofitamab induces NSTs after 82 days (range, 1∼322), while the induction time for tarlatamab is 16.7 days (range, 1∼141). This variability underscores the importance of monitoring adverse reactions based on the specific drug being administered in clinical settings.

This study has several limitations. First, the analysis relied exclusively on the FAERS database, which may have excluded certain neurological adverse reactions from the statistics, leading to incomplete datasets. Second, some BiTE drugs have been marketed for a relatively short duration, resulting in limited reports of adverse reactions. Third, reports submitted by non-medical professionals may introduce diagnostic biases, and the description and recording of neurological adverse events cannot be precisely graded. Additionally, as the FAERS database relies on spontaneous reporting systems, it is susceptible to biases and errors in the reported information. These limitations underscore the challenges associated with relying solely on self-reported data. Future research should aim to integrate adverse reaction reporting systems with patients’ electronic health records or patient registration systems to enhance the accuracy and comprehensiveness of the results.

## 5 Conclusion

Although BiTEs have revolutionized the treatment landscape for hematological malignancies, their associated neurological toxicities remain a major clinical challenge. Advances in molecular engineering, biomarker-driven interventions, and innovative delivery systems are paving the way for safer and more effective BiTE therapies. Future research should focus on the development of predictive models for neurotoxicity risk assessment, exploration of central nervous system-penetrant immunomodulators, and integration of multi-omics approaches to elucidate the complex interplay between T cell activation and neuroinflammation. Addressing these challenges will be critical to realize the full potential of the next generation of BiTEs in both hematologic and solid tumors, offering durable remissions without compromising neurological integrity.

## Data Availability

The original contributions presented in the study are included in the article/supplementary material, further inquiries can be directed to the corresponding authors.
